# Exploring the chaotic future and the deterministic past of the Solar System: an interview with Jacques Laskar

**DOI:** 10.1093/nsr/nwag002

**Published:** 2026-01-05

**Authors:** Mingsong Li, Weijie Zhao

**Affiliations:** School of Earth and Space Sciences at Peking University, China; news and science editor of NSR based in Beijing, China

## Abstract

Jacques Laskar (1955–) is a preeminent French astronomer and celestial mechanician whose work has fundamentally reshaped our understanding of the Solar System’s long-term dynamics and Earth’s climate history. As a Research Director at the CNRS (French National Centre for Scientific Research) and at the Paris Observatory, he is widely known for his 1989 numerical demonstration that the Solar System’s long-term evolution is chaotic and therefore cannot be precisely predicted. He is also renowned for developing numerical astronomical solutions that underpin astronomically calibrated timescales. For these transformative contributions, he has received prestigious accolades, and an asteroid has been named after him. In recent years, he has focused on the AstroGeo project, which aims to invert geological records to infer past orbital parameters and reconstruct the precise planetary motions across large spans of geological time.

In December 2025, NSR spoke with Prof. Laskar in Beijing. During the conversation, he shared insights on the Solar System’s past, present and future, discussed his current research and reflected on his unusual career path, from a high-school teacher to a leading scientist.

## Teaser

NSR spoke with Jacques Laskar, a preeminent French celestial mechanician whose work has fundamentally reshaped our understanding of the Solar System’s long-term dynamics and Earth's climate history.

## THE CHAOTIC NATURE OF THE SOLAR SYSTEM

***NSR*:** When you look at the Solar System today, with all its structure, history and fragility, what picture comes most vividly to your mind?

***Laskar*:** For me, the current structure of the Solar System is the outcome of a self-organization process. When the system was first formed, there were many planets and the system was very unstable. Collisions between planets occurred and the system became more stable after each collision, because each collision decreased the system’s eccentricity and the possibility of orbit intersections. The last collision in the Solar System caused the formation of the Moon: Earth collided with a Mars-sized body in an orbit between Mars and Earth. In the end, the system stops at the edge of instability. Collisions are no longer possible or only possible on a much longer timescale.

Based on the long-term dynamics analysis of the Solar System, I proposed in 1994, before the first extrasolar planetary system was discovered, that most planetary systems would be in such a state of marginal stability, which means that collisions are possible, but only on the timescale that is comparable to the age of the system. This picture also means that the system is full, which means that you cannot add an additional planet. If you add an additional planet, collisions will occur very rapidly.

***NSR*:** How does our understanding of our own planetary system inform the way we search for habitable worlds around other stars?

***Laskar*:** In 1993, I showed that, without the Moon, the axis of Earth would no longer be stable; it can move chaotically between 0° and 90°. This means that having a planet in the habitable zone is not enough to make an Earth-like world. It would very likely be a planet with no stable obliquity. So it is not so easy to find a planetary system such as ours.

**Figure 1:**
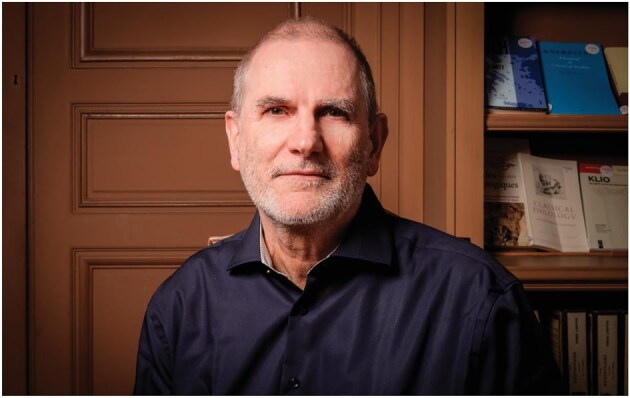
Prof. Jacques Laskar demonstrated that the Solar System’s long-term evolution is chaotic and cannot be precisely predicted. *(Photo: Courtesy of the interviewee; © Académie des sciences—Mathieu Baumer.)*

Another issue is that, right now, because of observational bias, people are looking for exoplanets around small stars. But the habitable zone of a small star lies close to the star and, in that case, all the planets are tidally locked, which means they always present the same face to the star, as the Moon always presents the same face to Earth. This does not mean that life is impossible there, but it will not be at all like life on Earth in our planetary system.

***NSR*:** Over the centuries, humanity’s view of the Solar System has transformed dramatically. What have been the key steps or turning points in this scientific journey?

***Laskar*:** That is a long story, but I will try to summarize it. Kepler published *Astronomia nova* in 1609, proposing that the planets were moving on fixed elliptical orbits around the Sun. In this theory, there was no problem of stability.

The question of stability was first raised by Newton after he proposed the law of gravitation in 1687, for, if all the planets attract each other, their orbits will be disturbed. Newton’s answer to this question was that God had to come from time to time to put the planets back onto their right paths. So, in the eighteenth century, questioning the stability of the Solar System was like questioning the power of God.

This question was practically solved by Laplace and Lagrange at the end of the eighteenth century. They demonstrated that, at first order, the sizes of the orbits do not change under mutual perturbations. The shape of the ellipses changes, they rotate slowly, but the orbit deformations are not enough to allow collisions. The Solar System thus recovered its state of everlasting stability.

And again, one century later, at the end of the nineteenth century, a big step was made by the French mathematician Henri Poincaré. He showed that the three-body problem is not integrable, which means you cannot find an analytical solution. He showed that there could exist very complex motions—what we now call chaotic motion, in which uncertainty can increase exponentially. But, although Poincaré showed the occurrence of chaotic behavior, he did not really think this would apply to the Solar System. He believed that, for the Solar System, dissipative effects such as tides would dominate and keep the system stable.

Then, between 1954 and 1963, the Russian mathematicians Kolmogorov and Arnold, and Swiss mathematician Moser demonstrated that, despite the system not being integrable, for sufficiently small planetary masses, there could exist isolated solutions for the planetary motions, in which the orbits are quasiperiodic and thus stable over infinite time. But, as shown by Michel Henon, this result is only valid for extremely small planetary masses—even smaller than the mass of an electron—and thus it cannot offer proof of the stability of the Solar System.

The next step was what I did in 1989. I built dedicated computer algebra software and pushed Laplace and Lagrange’s analytical computation to high order; combined with numerical integration, I performed simulations of the full Solar System for 200 million years (Myr). The result showed that the motion is chaotic, not quasiperiodic as previously thought. The chaotic behavior manifests as an exponential increase in uncertainty, increasing by a factor of 10 every 10 Myr. So, if you have a 15-meter uncertainty at present, after 10 Myr, it is just 150 meters, which is negligible. But, after 100 Myr, it becomes 150 million kilometers (15 × 10^10^ m)—the size of the Sun–Earth distance, which is a huge difference.

We don’t know yet the probability of a collision involving Earth.

—Jacques Laskar

Later, in 2008, I showed that the planets can indeed collide. Actually, I had proposed this idea in 1994, but a sophisticated method and analytical averaged equations were used. This was not convincing for all. By 2008, when computers became powerful enough, I addressed the problem by using a brute-force approach and the paper was published in *Nature* [J Laskar *et al. Nature* 2009; **459**: 817–9]. I basically recovered the results obtained in 1994 and proved that Earth can collide with Mercury, Mars or Venus, within 5 × 10^9^ years (5 Gyr).

***NSR*:** Looking forward, what aspects of Solar System dynamics do you believe we are closest to unlocking and what aspects remain the most elusive?

***Laskar*:** Now we know that the Solar System is chaotic and the probability of planetary collisions is in the order of 1% over 5 Gyr. But this mainly concerns the most unstable planet, Mercury. We don’t know yet the probability of a collision involving Earth, which is much smaller than the one involving Mercury. I think it’s possible to calculate that probability, but it requires some time.

Another question is to reconstruct the past evolution of the planetary orbits over the past 4 Gyr, from the present back to the time at which the Solar System had reached roughly its present configuration. We can describe it in a statistical sense, but, at present, due to its chaotic behavior, we cannot recover the past orbits of the planets beyond ∼60 million years ago (Ma).

## QUEST FOR A DETERMINISTIC SOLAR SYSTEM PAST

***NSR*:** Why is it difficult to reconstruct the past of our Solar System beyond 60 Ma?

***Laskar*:** If you have the current parameters of the planets with infinite precision, as well as a numerical integration method with infinite precision, you will be able to obtain a unique solution for the planetary orbits over infinite time. But, in practice, you cannot do that.

In 2011, I demonstrated that the main limitation was the perturbations caused by the largest minor planets, and in particular Ceres and Vesta, which themselves have chaotic motion, with a divergence by a factor of 10 in <50 000 yr. This uncertainty limits planetary predictions to ∼60 Ma.

This limit is very hard to beat. Indeed, even if we improve everything, the accuracy of the initial conditions and the accuracy of the model, by a factor of 1000, going from meter precision to millimeter precision, we would gain only 150 000 yr in the predictions, pushing the boundary from 60 to 60.15 Ma. So the 60-Ma limit is really intrinsic, due to the nature of the interactions, and not only due to the limited precision in the initial conditions.

However, we can overcome this limit if we have some additional information on the past positions of the planets. Unfortunately, there were no astronomers among the dinosaurs that recorded the planetary positions; fortunately, such information was imprinted in the sedimentary record. Building on work from the 1910s and 1920s, Milutin Milanković a Serbian scientist, culminated his orbital-climate calculations in 1941 with the *Canon of Insolation*, showing how orbital variations reshape the distribution of solar radiation and thereby affect past climates. Since the pioneering work of Hays, Imbrie and Shackleton (1976), we have known that this astronomical signal can be recognized in sediments.

**Figure 2:**
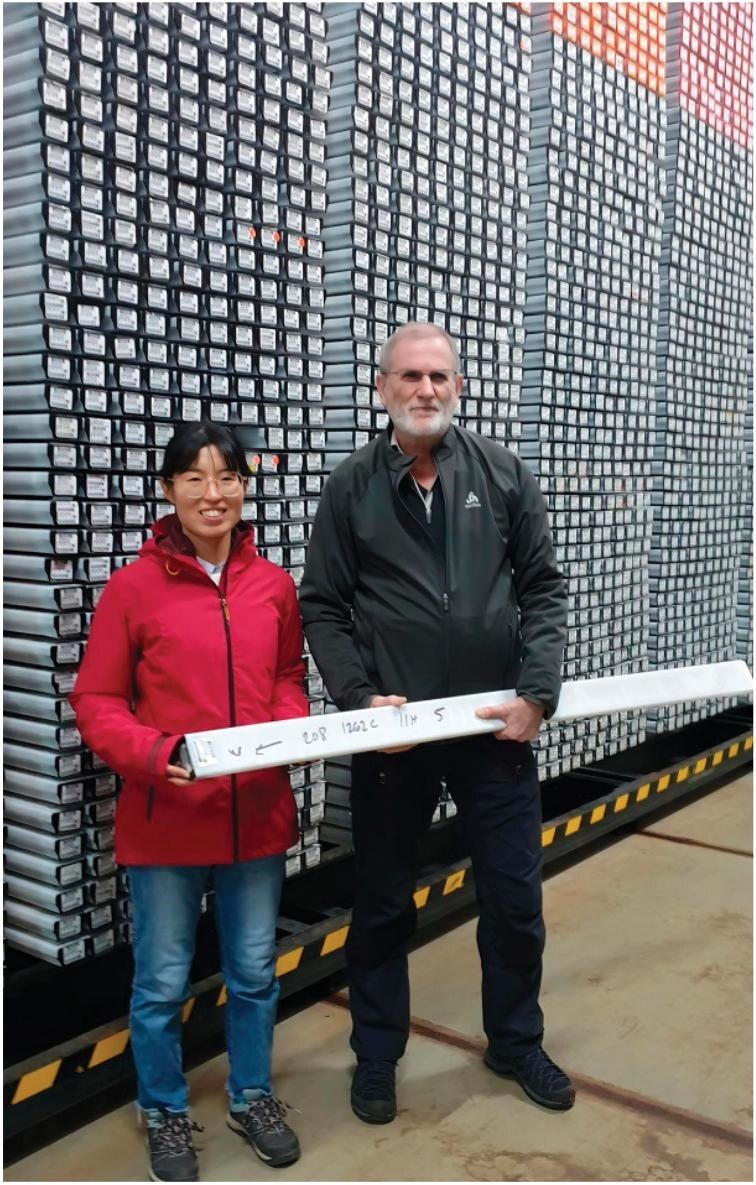
Prof. Jacques Laskar and postdoctoral researcher Yujing Wu pose at the Bremen Core Repository in Germany, a facility holding Ocean Drilling Program cores that can be used to reconstruct orbital parameters. *(Photo: Courtesy of the interviewee; taken by Dr. Thomas Westerhold of the University of Bremen.)*

Now we are trying to use this sedimentary information to constrain the past evolution of the Solar System. This is a very difficult task, as it requires the best sedimentary sequences, which are not so numerous. Very often, over a given time span, there is only one available sedimentary record, and that is not enough. We need several high-quality records to screen out the noise introduced by various processes and to get confidence in the recovered astronomical signal.

So what we really need now, therefore, are long sequences of sedimentary records of the highest quality. I have some hope that, in the next 10 years, we will recover the unique past of the Solar System of the last 100 Myr, and possibly extend this reconstruction to the entire Mesozoic (∼250 Myr).

***NSR*:** Could you briefly introduce the AstroGeo project? After several years of work, in what ways has it transformed our understanding of Solar System evolution and past climate?

***Laskar*:** For more than three decades, geologists have used my orbital solutions to establish timescales for their geological records. Actually, many years ago, I began to think that the astronomical information imprinted in the sedimentary record needs to be used as astronomical observations and help constrain the orbital solution to go beyond 60 Ma. That is why I set up the AstroGeo project.

I did not start this project until 2020, because it is highly interdisciplinary work. I was a little bit reluctant to push some students into it at first, as I worried it might be difficult for them to find positions after graduation.

When I finally decided to do it, I applied for both a European funding project and a French funding project, and I got both. I was actually quite surprised by the enthusiastic responses from the 11 reviewers of the AstroGeo project. I did not expect to receive such unanimous support.

I outlined several scientific goals in the proposal, and we have indeed gone much further than what I wrote in at least two directions.

The first is about the Earth–Moon evolution. Apollo samples showed that the age of the Moon is ∼4.5 billion years, while the Moon’s age calculated from its present recession speed is only 1.5 billion years. This discrepancy has confused everyone for half a century. To my surprise, we not only make progress in this direction, as I wrote in the proposal, but actually solved the problem in some sense. We found a coherent scenario that reconciles the Moon’s age with its present orbital dynamics [Farhat *et al. Astron Astrophys* 2022; **665**: L1]. While this may not be a definitive final answer, it has made a big difference, and is now widely adopted as a reference in the field, particularly by geologists searching for billion-year-old sediments.

The other major achievement of AstroGeo is that we devised a new method, AstroGeoFit, that is able to work out the astronomical information from the geological sediment record without the need for an astronomical solution [Hoang N *et al. Paleoceanogr Paleoclimatol* 2025; **40**: e2024PA005021]. Now we can derive the orbital motion of Earth in a quantitative way from the data and that gives us hope to reconstruct a longer history. In addition, together with the publication of the paper, we have released AstroGeoFit as an open-source Python package that allows everyone to implement our method (www.astrogeo.eu).

***NSR*:** Now you have thousands of astronomical solutions, do you want to find the best one among them?

I applied for both a European funding project and a French funding project, and I got both … I did not expect to receive such unanimous support.

—Jacques Laskar

***Laskar*:** Because of the system’s chaotic behavior, we now have thousands of solutions, which are very different after 60 Ma. We do want to find the solution that represents the actual past of the Solar System. That is a real challenge. Predicting the future is easy because nobody will know. But, for the past, we may find evidence to judge what is right or wrong.

***NSR*:** The Solar System is embedded in a much larger galactic environment. Can we calculate the external influences on our Solar System?

***Laskar*:** The Solar System is embedded in the Galaxy. We know that the Sun moves within it, following an orbital motion with a period of ∼200 Myr and an oscillatory motion with a period of ∼70 Myr. But, even though we have the Gaia satellite and some other observations, we still don’t know the precise orbital motion of the Sun in the Galaxy. Thus, we still cannot tell how these motions were imprinted in the sedimentary record.

I have tried to tune a specific model for the motion of the Sun in the Galaxy to fit the geological sea-level record, but it is still conjectural until we know better about the motion of the Sun.

## THE METHODOLOGY OF CELESTIAL MECHANICS

***NSR*:** Do you foresee artificial intelligence (AI) and other new computation methods playing a transformative role in celestial mechanics and planetary dynamics?

***Laskar*:** I think AI will play a role, especially in the analysis of sedimentary data. We are already using some of the AI methods in AstroGeoFit. In celestial mechanics, I know there are also some attempts to incorporate AI, but I am not very familiar with them.

***NSR*:** If you suddenly had 10 times the computational resources, what would be the very first question you would try to answer?

***Laskar*:** I would say that I have never been limited by computational resources, because the most difficult aspect was how to approach the problems. My first demonstration of the chaotic nature of the Solar System used only 100 hours of computing time. And, for chaotic systems with exponential divergence, 10 times more computing power is nothing, and it would not lead to much progress.

***NSR*:** Most of your work has involved computational simulations, but have you ever done experiments?

***Laskar*:** Yes. I studied the rotation of Venus in around 2000 with my PhD student at the time, Alexandre Correia. We tried to model the atmospheric thermal tides, but our model was not fully satisfactory. Several years later, using a good meteorological station in my garden, I recorded the barometric pressure every minute for a whole year. After that, I folded all the data into a single day to average out the meteorological fluctuations and what remained was a very smooth curve that represents the actual atmospheric solar tides. I asked another PhD student to work on these curves and we built a better model [Auclair-Desrotour *et al. Astronomy and Astrophysics* 2017; **603**: A108].

I focus on problems, not methods. When I want to understand something, I will use any method I can find to solve it.

## RETROSPECT AND ADVICE

***NSR*:** Has your scientific career unfolded smoothly, or have there been pivotal challenges along the way?

***Laskar*:** In fact, my academic path was not straightforward. After getting my first master’s degree in mathematics, I became a high-school teacher and found it to be a nice job. But, after some time, I realized that I would not do this for my entire life, and I started to studying psychology part-time. I enjoyed it a lot and decided to become a psychiatrist. I was able to pursue psychology degrees while teaching at high school at the same time. However, to enroll in medical studies, I needed to study full-time. I resigned from my teaching position but needed to earn a living. I thought I could give a few well-paid mathematics classes to earn a little money, but such a position requires the Agrégation in mathematics (the highest-level national teaching certification in France). So I decided to get the Agregation first and returned to the study of mathematics, and that was when I found research enjoyable. After I got the Agregation, I went on to earn my second master’s degree in astronomy and celestial mechanics, and then my PhD.

I had been a high-school teacher for three years, but, because I started university early and completed my PhD in just 18 months, I was only 29 when I finally earned my doctorate.

***NSR*:** You completed your PhD in just 18 months—that is really remarkably efficient.

***Laskar*:** Yes, because I was highly motivated. When I worked on my second master’s degree, I was struck by two things. One was the fact that the three-body problem was not integrable. The other was that I found that there was a gap between what I could see from my mathematical background and what was taught at the time in celestial mechanics, which was rather old-fashioned. I really wanted to bridge that gap.

Celestial mechanics was a somewhat dusty field at the time and many people believed there was nothing new to discover there. But I was genuinely interested in this field and

Celestial mechanics was a somewhat dusty field at the time and many people believed there was nothing new to discover there. But I was genuinely interested in this field and I thought, ‘Okay, I will take the broom and sweep the dust away.’

—Jacques Laskar

I thought, ‘Okay, I will take the broom and sweep the dust away.’ Now, people say the opposite and tell me that I am lucky to work in such a stimulating field.

***NSR*:** Did your experience as a high-school teacher help with your research?

***Laskar*:** Yes. When you are teaching young children, you have to get them interested. That is not so easy, especially in mathematics. So I learned to take into account the audience when giving a talk. That is something I am still trying to do.

***NSR*:** What advice would you share with young researchers?

***Laskar*:** From my own experience, I would say that you should find something that truly motivates you. If you do, you will not worry too much about what people say or whether it is considered one of the fanciest topics at the time. What matters most is the willingness to explore beyond familiar paths.

I sometimes compare scientific research to mountaineering. People can be very competent climbers on indoor walls, or even outdoors in well-protected environments. But another thing is to go to the mountains—to places where people did not even think it was possible to go. You may not need to take a very difficult path. In science, daring to venture onto such mountains and trying to find a route to the top is one of the most important aspects of doing good research.

## Notes

Mingsong Li is an Assistant Professor in the School of Earth and Space Sciences at Peking University, China; Weijie Zhao is a news and science editor of NSR based in Beijing, China.

